# Effects of electroacupuncture therapy for Bell’s palsy from acute stage: study protocol for a randomized controlled trial

**DOI:** 10.1186/s13063-015-0893-9

**Published:** 2015-08-25

**Authors:** Zhi-dan Liu, Jiang-bo He, Si-si Guo, Zhi-xin Yang, Jun Shen, Xiao-yan Li, Wei Liang, Wei-dong Shen

**Affiliations:** Department of Acupuncture, Baoshan Hospital of Integrated TCM and Western Medicine, Shanghai, 201999 China; Department of Acupuncture, Shuguang Hospital, Shanghai University of Traditional Chinese Medicine, Shanghai, 201203 China; Department of Medical Imaging, Baoshan Hospital of Integrated TCM and Western Medicine, Shanghai, China; Department of Neurology, Baoshan Hospital of Integrated TCM and Western Medicine, Shanghai, 201999 China; Department of Gerontology, Baoshan Hospital of Integrated TCM and Western Medicine, Shanghai, 201999 China; Department of Neurology, Third People’s Hospital, Shanghai Jiaotong University, Shanghai, 201999 China

**Keywords:** Acupuncture, Bell’s palsy, Electromyography, F Wave test, Magnetic resonance imaging

## Abstract

**Background:**

Although many patients with facial paralysis have obtained benefits or completely recovered after acupuncture or electroacupuncture therapy, it is still difficult to list intuitive evidence besides evaluation using neurological function scales and a few electrophysiologic data. Hence, the aim of this study is to use more intuitive and reliable detection techniques such as facial nerve magnetic resonance imaging (MRI), nerve electromyography, and F waves to observe changes in the anatomic morphology of facial nerves and nerve conduction before and after applying acupuncture or electroacupuncture, and to verify their effectiveness by combining neurological function scales.

**Methods/Design:**

A total of 132 patients with Bell’s palsy (grades III and IV in the House-Brackmann [HB] Facial Nerve Grading System) will be randomly divided into electroacupuncture, manual acupuncture, non-acupuncture, and medicine control groups. All the patients will be given electroacupuncture treatment after the acute period, except for patients in the medicine control group. The acupuncture or electroacupuncture treatments will be performed every 2 days until the patients recover or withdraw from the study. The primary outcome is analysis based on facial nerve functional scales (HB scale and Sunnybrook facial grading system), and the secondary outcome is analysis based on MRI, nerve electromyography and F-wave detection. All the patients will undergo MRI within 3 days after Bell’s palsy onset for observation of the signal intensity and facial nerve swelling of the unaffected and affected sides. They will also undergo facial nerve electromyography and F-wave detection within 1 week after onset of Bell’s palsy. Nerve function will be evaluated using the HB scale and Sunnybrook facial grading system at each hospital visit for treatment until the end of the study. The MRI, nerve electromyography, and F-wave detection will be performed again at 1 month after the onset of Bell’s palsy.

**Trial registration:**

Chinese Clinical Trials Register identifier: ChiCTR-IPR-14005730. Registered on 23 December 2014.

## Background

Acupuncture therapy is widely used for the treatment of Bell’s palsy in China and throughout the world [1–4]; however, its effectiveness is still debatable because conclusions drawn from Cochrane reviews [5, 6] and meta-analyses [7, 8] do not support its clinical application, whereas clinical trials tend to prove its efficacy [9, 10]. Except for some defects in the protocol design and implementation, different manipulations among Chinese and foreign physicians, and the lack of powerful evidence of objective detection and assessment besides the neurological function scale, are very important reasons for the conflicting opinions.

The detectable pathological changes of peripheral facial paralysis (including Bell’s palsy) are facial nerve edema and bioelectric conduction disorders. The former can be detected and evaluated using magnetic resonance imaging (MRI) [11–15], the latter using more sensitive neural electrophysiological methods [16].

Treatments for Bell’s palsy should differ according to the stage of the disease [17], and the most important factor in treatment is not the kind of manipulation but when to initiate the intervention, with the goal being to start in the acute stage within 7 days of palsy onset [18–20]. Currently, electroacupuncture is widely used in the treatment of Bell’s palsy, although there is inadequate evidence-based support, especially for use in the acute stage. On the one hand, some clinical experiences have revealed that an early and appropriate electroacupuncture therapy shortens the disease course and improves the curative effect [9, 21]. On the other hand, there are beliefs that electroacupuncture stimulation is not appropriate for patients in the acute stage of Bell’s palsy and that it is more likely to aggravate facial nerve edema and bioelectric conduction disorders. Thus, the efficacy of electroacupuncture for treating Bell’s palsy starting from the acute stage is yet to be investigated.

The purpose of this study is to investigate the effects of electroacupuncture therapy starting from the acute stage of Bell’s palsy. Symptom improvements will be evaluated by using the internationally accepted House-Brackmann (HB) Facial Nerve Grading System and the Sunnybrook facial grading system. Changes in facial nerve edema and nerve conduction will be analyzed by using MRI and neural electrophysiology techniques. The aim of the study is to provide a more reliable scientific basis for evaluating the effectiveness of electroacupuncture therapy for Bell’s palsy by combining clinical symptoms with physiology and electrophysiology findings.

## Methods/Design

### Study design

**Fig. 1:**
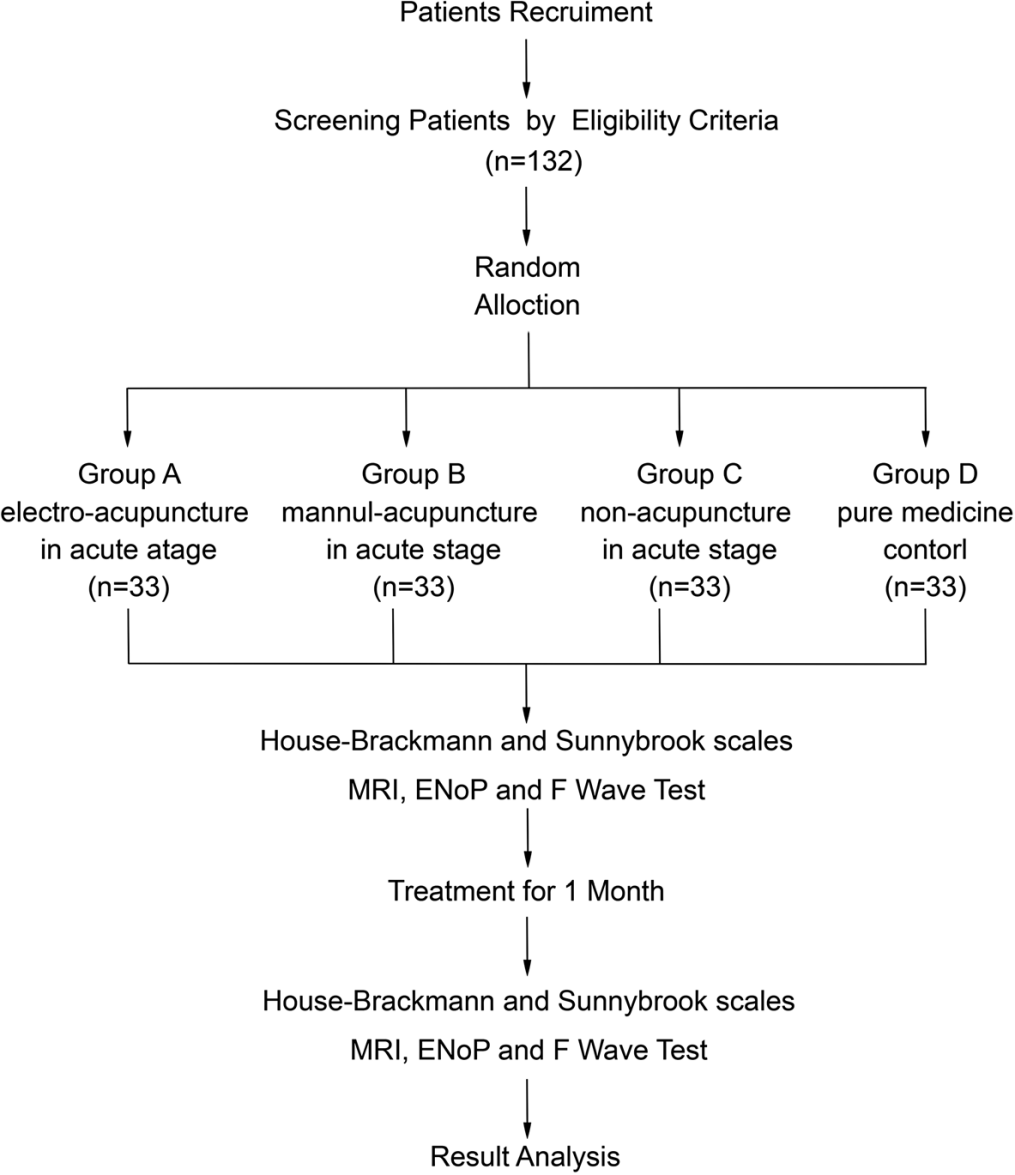
Flowchart of the study. *ENoP* neural electrophysiological

The present study is a randomized controlled clinical study (see Fig. 1).

### Ethics

The trial will be carried out in accordance with the Declaration of Helsinki. Written informed consent will be obtained from each participant. This study is approved by the Medical Ethics Committee Board of Baoshan Integrated Traditional Chinese Medicine and Western Medicine Hospital of Shanghai (2014XBN02).

### Patients

Both outpatients and inpatients in the departments of acupuncture, neurology, and gerontology of Baoshan Integrated Traditional Chinese Medicine and Western Medicine Hospital of Shanghai, China, are being selected for inclusion. Male and female patients ranging in age from 18 to 60 years old are being enrolled. All the patients are being randomized into group A (electroacupuncture in the acute stage), group B (manual acupuncture in the acute stage), group C (non-acupuncture in the acute stage), or group D (medicine control group).

### Sample size calculation

Count data of the parallel controlled trial (1:1) will be analyzed statistically using a two-sided superiority test where α = 0.05, power = 90 %. The formula used for calculation is as follows:

$$ n=\frac{{\left({Z}_{1-a}+{Z}_{1-\upbeta}\right)}^2\times \left({\sigma}_1^2+{\sigma}_2^2\right)}{\delta^2} $$

It is assumed that the change in the standard deviation of the Sunnybrook facial grading system score before and after the treatment in the control group is 10 points and that that in the treatment group is 18 points. A change will be considered clinically significant if the change of Sunnybrook scale score before and after the treatment in the treatment group is 13 points higher than that in the control group. The estimated sample size is arrived at as follows:

$$ n=\frac{{\left({Z}_{1-a}+{Z}_{1-\upbeta}\right)}^2\times \left({\sigma}_1^2+{\sigma}_2^2\right)}{\delta^2}=\frac{{\left(1.96+1.28\right)}^2\times \left({10}^2+{18}^2\right)}{13^2}\approx 26.33 $$

It is assumed that the change in the standard deviation of HB scale score before and after the treatment in the control group is 1 point and that that in the treatment group is 3 points. A change will be considered clinically significant if the change of HB scale score before and after the treatment in the treatment group is 2 points higher than that in the control group. The estimated sample size is arrived at as follows:

$$ n=\frac{{\left({Z}_{1-a}+{Z}_{1-\upbeta}\right)}^2\times \left({\sigma}_1^2+{\sigma}_2^2\right)}{\delta^2}=\frac{{\left(1.96+1.28\right)}^2\times \left({1}^2+{3}^2\right)}{2^2}=26 $$

It is determined that there should be at least 27 patients in each group. The dropout rate is considered to be 20 %, and thus at least 33 patients should be enrolled in each group. Hence, a total of 132 patients are required to conduct this study.

### Inclusion criteria

Patients with Bell’s palsy who meet the following inclusion criteria will be enrolled in the study: unilateral facial paralysis; age between 18 and 60 years; Bell’s palsy onset within 2 days; HB scale score above grade III; normal superficial and deep sensations, muscle strength and muscle tension in limbs, with negative pathological signs; and absence of heart failure, abnormal liver function, and other severe complications of diabetes mellitus.

### Exclusion criteria

Patients meeting any of the following criteria will be excluded from the study: pregnancy or breastfeeding; woman of childbearing age who is unwilling to use contraceptives during the study period; patients who received medicine or acupuncture therapy in other hospitals before this study; diabetes course ≥5 years, grade 3 hypertension ≥3 years, and hyperlipidemia ≥3 years; history of malignant tumor, sexually transmitted disease, renal or hepatic disease, gastric or duodenal ulcer, glaucoma, acute otitis, ipsilateral chronic otitis, tuberculosis, immunodeficiency syndromes, recent head injury, psychiatric disease, infectious disease, or any other condition that puts the person at risk of being influenced by the study treatment or that may affect the completion of the study; and participation in other clinical trials within 3 months.

### Elimination criteria

Patients with cerebral infarction, cerebral hemorrhage, or other brain tumors diagnosed on the basis of skull computed tomography or MRI and patients who refuse to sign the written informed consent or did not coordinate with the randomized enrollment will be excluded from the study.

### Randomization

Patients will be randomly assigned to one of the four treatment groups. Computerized randomization will be performed by an investigator who has no direct contact with the participants. The assessors will be blinded to the group allocation. The investigator performing the acupuncture intervention will not be intrinsically blinded; however, the investigator will not be allowed to communicate with the participants or the assessors about the treatment procedures and outcomes. A sealed envelope containing an allocation sequence number for each patient will be opened right after each patient is verified to meet the eligibility criteria and has signed the informed consent form. If any error or disclosure with regard to randomization occurs, a new randomization sequence will be generated starting from the problematic serial number and applied to subsequent patients.

### Interventions

#### Acupuncture therapy

Acupuncture will be performed by specialists in traditional Chinese medicine, and the protocol will follow the details of the Standards for Reporting Interventions in Clinical Trials for Acupuncture 2010 checklist [22, 23], as shown in Table 1.

**Table 1:** Details of acupuncture intervention

		Description
Acupuncture rationale	Style of acupuncture	Traditional Chinese medicine
	Rationale for treatment	Acupuncture has been historically used to treat facial palsy. Additionally, it is known to be a safe treatment used in a wide range of symptoms caused by Bell’s palsy
	Extent to which treatment varied	The subjects of the intervention group all receive the same acupuncture or electroacupuncture treatment
Details of needling	Number of needle insertions per subject per session	10
	Names of the insertion points (uni- or bilateral)	GB14, TE23, Qianzheng, LI20, SI18, ST4 (unilateral, affected side), LI4, LR3 (bilateral)
	Depth of insertion	10–30 mm (exact depth shown in Table 2)
	Response sought	*Deqi*
	Needle stimulation	Some in electric stimulation, others in manual stimulation (exact details are in the text)
	Needle retention time	20 minutes
	Needle type	0.30 mm (diameter) × 25 mm (length) disposal needle (Huatuo Acupuncture, Suzhou, China)
Treatment regimen	Number of treatment sessions	12
	Frequency and duration of treatment sessions	3 sessions/wk for 4 wk
Other components of treatment	Details of other interventions administered to the acupuncture group	No other interventions
	Setting and context of treatment	All subjects are informed that they will receive acupuncture or electroacupuncture treatment, which can potentially reduce Bell’s palsy symptoms; however, the non-acupuncture control group would have to complete the evaluations during the first week before receiving the same treatment as the acupuncture group.
Practitioner background	Description of participating acupuncturists	Specialists in traditional Chinese medicine with at least 3 years of practice in acupuncture
Control or comparator interventions	Rationale for the control or comparator in the context of the research question	Non-acupuncture control is used as a control because sham acupuncture cannot be a substituted for a physiologically inert placebo, and Western medicine is recommended in the guideline.
	Precise description of the control or comparator	The Western medicine control group forms a positive control and completes the evaluations during the first week after randomization.

### Acute stage

Patients in groups A and B will receive acupuncture therapy at the Cuanzhu (BL2), Yangbai (GB14), Sizhukong (TE23), Qianzheng, Yingxiang (LI20), Quanliao (SI18), Dicang (ST4), Hegu (LI4), and Taichong (LR3) points. The acupuncture points are identified according to the method of point location issued by the World Health Organization (WHO). The acupuncture needles will be inserted straightly or obliquely with a depth of 10–30 mm to bring about the desired sensation of *deqi* (see Table 2), and the needles will be left in place for 20 minutes. The acupuncture therapy will be performed once every 2 days. Accordingly, patients in group A (electroacupuncture in the acute stage) will receive electroacupuncture therapy at the Yangbai (GB14)-Sizhukong (TE23), Quanliao (SI18)-Yingxiang (LI20), and Qianzheng-Dicang (ST4) points (2 Hz, density wave, 20 minutes) once every 2 days. Patients in group C will not receive acupuncture therapy.

**Table 2:** Acupuncture points and needle insertion procedures

Acupuncture point	Direction	Depth (mm)
GB14 (yangbai, affected side)	Transversely toward geisoma	20–30
SJ23 (sizhukong, affected side)	Obliquely toward partes temporalis	20–30
Qianzheng (extra point, affected side)	Perpendicular to the skin	20–30
LI20 (yingxiang, affected side)	Obliquely along nasolabial sulcus toward the root of the nose	20–30
SI18 (quanliao, affected side)	Perpendicular to the skin	20–30
ST4 (dicang, affected side)	Transversely toward ST6	10–20
LI4 (hegu, bilateral)	Perpendicular to the skin	20–30
LR3 (taichong, bilateral)	Perpendicular to the skin	20–30

### Stable and recovery stages

Patients in groups A–C will receive straight or oblique acupuncture with a depth of 10–30 mm at the Cuanzhu (BL2), Yangbai (GB14), Sizhukong (TE23), Qianzheng, Yingxiang (LI20), Quanliao (SI18), Dicang (ST4), Hegu (LI4), and Taichong (LR3) points (identified according to the method of point location issued by the WHO) to bring out the desired sensation of *deqi* from the eighth day after palsy onset. The needles will be retained for 20 minutes. The electroacupuncture will be performed once every 2 days. Accordingly, patients in groups A–C will receive electroacupuncture therapy at the Yangbai (GB14)-Sizhukong (TE23), Quanliao (SI18)-Yingxiang (LI20), and Qianzheng-Dicang (ST4) points (2 Hz, density wave, 20 minutes). All patients will be given therapies until the endpoint of complete recovery or study withdrawal.

### Western medicine treatment

Patients in each group will be given oral administration of prednisone (30 mg once daily for 5 days and then 5 mg daily until study withdrawal), mecobalamin (0.5 mg thrice daily), and fursutiamine (50 mg thrice daily) until complete recovery. Group D serves as the medicine control group, which will not be given any acupuncture or electroacupuncture therapy throughout the trial.

### Outcome

To reduce the outcome assessment bias, the investigators will be given specific training for facial grading, and both the Sunnybrook and HB scales will be used for outcome evaluation.

### Primary outcome

Evaluation will be performed using facial paralysis evaluation scale (HB and Sunnybrook scales). Measurements will be performed at the above-mentioned time points and at the first month after therapy by three physicians, and the averaged value will be recorded. Meanwhile, the time for the first change in the score after the start of treatment will be labeled. The period from palsy onset to the first change in the facial paralysis evaluation scale score and the patient’s overall recovery period will be recorded, and comparison between the interventional groups and the control group will be conducted.

### Secondary outcomes

#### MRI examinations

The MRI examinations (MAGNETOM version 3.0T MRI Scanner using head matrix coils; Siemens Medical Solutions, Erlangen, Germany) will be performed within 7 days and at 1 month after the onset of Bell’s palsy. The conventional MRI protocol for this study consists of the following sequences. Three-dimensional constructive interference in the steady state will be performed in an axial orientation with the following parameters: 18-cm field of view (FOV), repetition time/echo time (TR/TE) of 8.58/3.91 ms, 64 sections of 0.5-mm thickness, with a matrix of 320×320 pixels, flip angle 50 degrees, bandwidth of 460 Hz, and acquisition time of 6 minutes, 12 seconds. A volume-interpolated body examination will be performed in precontrast and contrast T1 with the following parameters: 18-cm FOV, TR/TE of 20/3.69 ms, 32 sections of 1-mm thickness, with a matrix of 320×320 pixels, flip angle 12 degrees, bandwidth of 130 Hz, and acquisition time of 3 minutes, 4 seconds. Gadolinium-diethylenetriamine pentaacetic acid (contrast medium) will be administered intravenously at a dosage of 0.1 mmol/kg.

#### Facial nerve electromyography

The surface recording electrodes will be placed on the orbicularis oculi muscle and the orbicularis oris. The reference electrode will be placed on the nasal bone. The ground electrode will be placed on one arm. Super strong stimulations will be applied to the stylomastoid foramen, and the latency and amplitude of the motor evoked potentials (M wave) at the initial location will be observed, first on the unaffected side and then at the same location on the affected side. The results of the two sides will be compared. Meanwhile, the R1/M-wave ratio in latency will be measured. The measurements will be performed within 7 days and at 1 month after palsy onset.

#### F-wave detection of facial nerve

The participants will be in supine position in a silent room with constant temperature, and the examination (normally right side first) will be performed after 5 minutes of relaxation. An electromyogram evoked potential test (Dantec Keypoint; Alpine Biomed, Skovlunde, Denmark) will be performed. The surface electrodes will be placed on the orbicularis oculi muscle and the depressor anguli oris muscle, and the stimulus (20 times of square wave with a time history of 0.3 ms, firing frequency of 1 Hz) will be applied on nervi auriculares anteriores. After the stimulating current strength is gradually increased to approximately 120–130 %, with a filtering range of approximately 100–5000 Hz and analysis duration of 50 ms, the presence of F waves in the affected and unaffected sides and the relevant data will be recorded. The measurements will be performed within 7 days and at 1 month after palsy onset.

### Statistical analysis

In this study, statistical analyses of data will be performed by professionals rather than investigators using intention-to-treat and per-protocol approaches. All demographic and clinical characteristics of the subjects (such as sex, age) will be processed based on descriptive analyses. Quantitative data will be presented as average, standard deviation, median value, and range. Qualitative data will be presented as the frequency and percentage.

For the primary outcome measurements, a repeated-measures multifactorial analysis will be performed to identify differences between groups (differences within each group based on time, and the effects of the interaction of the variables based on group) to compare the differences in HB and Sunnybrook score changes between the interventional and control groups based on time (baseline, every treatment time and 1 month after palsy onset). The period from the onset to the first change in facial paralysis evaluation scale score in and the patients’ overall recovery period will be compared using one-way analysis of variance (ANOVA) between all groups. For the secondary outcome measurements, a χ^2^ test will be used to compare the edema onset frequency of the facial nerve on the affected side. One-way ANOVA will be applied to identify differences in results of facial nerve electromyography and F-wave detection between groups. In cases of non-normally distributed data, the Kruskal-Wallis H test will be performed.

All adverse events reported during the study will be included in the clinical report, and the prevalence of adverse events will then be calculated. The percentage of participants with adverse events in each group will be calculated and compared using the χ^2^ test or Fisher’s exact test. Statistical analyses will be performed using IBM SPSS for Windows version 19.0 statistical software (IBM, Armonk, NY, USA). All the tests will be two-sided, and a *P* value <0.05 will be considered statistically significant.

## Discussion

It is recognized that prednisolone but not antiviral medicine shows significant short- and long-term positive treatment effects in patients with Bell’s palsy [24–29]. This treatment should be used routinely for patients with Bell’s palsy and should be considered in all of these patients irrespective of degree of palsy [30]. It is noticed in clinics that acupuncture therapy alone is not likely to achieve significant curative effect without prednisolone for patients with HB scale scores equal to or over grade V. So, prednisolone is given to treat Bell’s palsy conventionally nowadays.

This study is designed based on a treatment including giving prednisolone, which will be used in every group. The purpose is to compare the different results of intervention by electroacupuncture, acupuncture, and non-acupuncture in the acute stage, followed by electroacupuncture in subsequent stages. Thus, the protocol is more likely to be in accord with current clinical practice and to be more conducive to protecting patients’ rights, as well as avoiding delay in treatment owing to bias of study design to a maximum extent. Hence, it is more scientific and reasonable to adopt a treatment strategy of Western medicine plus acupuncture therapy or electroacupuncture in the acute stage of Bell’s palsy facial paralysis.

The greatest uncertainty in this study is the effect of acupuncture or electroacupuncture on duration of disease or the completeness of ultimate recovery. However, either positive or negative effects eventually will be used to illustrate the facts, facilitating development of a reasonable clinical strategy in the future.

The aim of this study is to provide evidence of the effects of electroacupuncture therapy on Bell’s palsy. The sample size estimation of the present study was based on previous clinical scale evaluation, and the obtained sample size might, to some extent, differ from previous electrophysiological studies owing to the difficulty in obtaining data regarding the latter, such as mean values of a healthy population and patients with Bell’s palsy. This may be one of the aspects affecting the ultimate data analysis of the present study. Hence, it might be necessary to expand the sample size during the study to obtain credible results.

## Trial status

Recruitment started in January 2015 and will be completed by the end of May 2017.

## Abbreviations

ANOVA: Analysis of variance
ENoP: Neural electrophysiological
FOV: Field of view
HB: House-Brackmann
MRI: Magnetic resonance imaging
TR: Repetition time
TE: Echo time
WHO: World Health Organization
